# The Future of St. Bartholomew's Hospital

**Published:** 1903-01-17

**Authors:** 


### HOSPITAL ADMINISTRATION.

### CONSTRUCTION AND ECONOMICS.

### THE FUTURE OF ST. BARTHOLOMEW'S HOSPITAL.

The Present Position.

Since our last issue the daily papers have been fairly full of the St. Bartholomew's proposal to issue an appeal for £300,000 to defray the cost of new buildings upon the present site at Smithfield. Most of the comments in the Press have been distinctly hostile to the proposal, and the *Westminster Gazette* went so far as to warn the Lord Mayor from taking a step "which would be remembered as a serious mistake in his Lord Mayoral record." The Governors of St. Bartholomew's Hospital maintain that the great point is that they are the City hospital, that the City provided them with the money over one hundred and fifty years ago when the present buildings were erected, and that they have a right to ask the City to supply the money now, either in the form of a capital sum of £300,000, or by way of new income amounting to £20,000 a year. The treasurer, in a letter to the *Times* on Monday, sets forth the facts we have summarised, but does not dispose of a single objection which has been raised against the proposals (*a*) to reconstruct and largely rebuild St. Bartholomew's Hospital on the existing site, and (*b*) to open a Mansion House Fund. Nevertheless the evidence forthcoming so far tends to show that the sale of the present site and the removal of the whole hospital to a new site in St. Luke's, about one mile distant from Smithfield, which is favoured by the surgical staff, would entirely obviate the necessity for any such appeal, lessen the risks to, and expedite the recovery of the patients admitted to treatment, and promote the best interests of the school, the hospital, and the public. In these circumstances it is essential that all the facts should be clearly understood and grasped by the public, the profession, and the Governors of St. Bartholomew's Hospital with a view to the wisest course being ultimately arrived at. We therefore propose to state them in order.

Beating the Air.

The immediate result of the criticisms which we made in our last number upon the present position has been to call forth an abundance of correspondence on the matter in the columns of the daily newspapers; but so far as we are able to discern no new facts of any importance have been forthcoming which can affect expert opinion on the question, and we think that so far from helping towards a sound conclusion much of the matter published merely confuses the issues. Our contemporary, the *Daily Mail*, has gone so far as to find much space in its columns for the opinions of one official of each of a great number of relatively small institutions, with the only possible result that progress and knowledge are not increased. It is mere cant to publish what purports to be the views of various hospitals when on examination the views are mainly those set forth by relatively small and unimportant establishments compared with the great general hospitals, which, for the most part, have so far shown a judicial reserve on the question which does them honour. For the rest, they are not hospitals at all, and are not entitled to be heard in a matter which cannot concern them even indirectly. St. Bartholomew's has as much or as little right as any other hospital in London to appeal to the public it serves for co operation and help in any direction whatever, that it may consider to be best calculated to promote its present and future welfare. To furbish up the opinions to the contrary of such relatively liliputian institutions as we have referred to, as if they should bear a mighty influence, is a mere farce. Such fulminations may occupy a column or so of space, but do not touch the discussion or affect the issue. In fact the collection and publication of such opinions must have a tendency, if it has any effect at all, to strengthen rather than to weaken the claims of St. Bartholomew's Hospital to public sympathy, while it necessarily diminishes the prestige of some institutions which have rushed into a controversy which may concern them little if at all. The great hospitals of London have as a rule wisely refrained from expressing any opinion on the matter; and although the impression has been conveyed by several newspapers that someone connected with the London Hospital is behind the present agitation against St. Bartholomew's Hospital, we cannot credit that the committee of any great hospital, much less that of the London, would commit itself to such a course of action. Indeed, we hope the authorities of the London Hospital, through its committee, will publish a disclaimer and definitely state that they are not actively opposing the projected action of St. Bartholomew's. If the British public once gets it into its head that a great voluntary hospital, whose excellent work has been universally and handsomely recognised, and which has in the past received, probably, more money from the public than any other hospital, is expending its energies and possibly its means in promoting an opposition to the efforts of the managers of another great hospital like St. Bartholomew's to make its work more efficient and helpful to the population of London, whatever other effects may result, we are confident, that such action must and will be resented, and that the damage accruing to the hospital which interferes may prove momentous and permanent. That is why we so much regret to see the great London Hospital's name mentioned so prominently and frequently in this connection. Everyone is wishful and anxious that the London Hospital Sustentation Fund this year shall be a success; but if this end is to be accomplished, the sooner a disclaimer is issued on its behalf the better it will be for the prospects of this Sustentation Fund.

The idea which is so prevalent, that an appeal for money from St. Bartholomew's Hospital would be injurious to the other hospitals by diverting subscriptions, will, we believe, prove to be incorrect. For the knowledge on this subject, obtained through the working of the central funds, has shown, that every additional effort of the kind which is made, by widening the area of givers, tends also to increase the sum in any given year received by all well-managed hospitals, and we think that in any case the above argument would be less applicable to St. Bartholomew's than to any other London hospital because of its position in the City, and the special interest that the institution consequently has in the minds of the many influential people in that quarter, who would willingly make a special effort to help St. Bartholomew's without in any way withdrawing their support from other hospitals.

### Questions for Decision.

In order to avoid all side issues and irrelevant matter let us here state what we consider to be the questions for decision, cleared from all cant, on which the future of St. Bartholomew's Hospital must depend:—

1. Is it justifiable for the Governors of St. Bartholomew's Hospital to expend hundreds of thousands of pounds on buildings on their present site in view of the fact that the value of the site is so enormous and its area so strictly limited?

2. What are the public aspects of the question: (*a*) In relation to the claims of the population, and (*b*) in relation to the safe and adequate treatment of the patients?

*Is the present site worth the proposed lavish expenditure?* It is admitted that no allowance can be made by the present scheme for future expansion. Has the hospital sufficient room for its present needs?

"Burdett's Hospitals and Asylums of the World" contains the views of the leading authorities which briefly come to this: With the exception of a few old hospitals in congested districts a hospital site should be sufficiently large to provide at least 1,000 square feet per bed. St. Bartholomew's, with the addition of the newly acquired land without deducting the area occupied by the medical school and other buildings, will only be able to supply 423 square feet per bed for 671 beds. It ought to contain, and on a new site would contain, 1,000 beds at least, a material factor, and the free superficial area would be at least 660 square feet per bed. Surely no further comment is necessary.

### The Safe and Adequate Treatment of Patients.

We gave figures in our last issue which showed that the population of the City of London had diminished by 76 per cent. in the last 40 years. If it can be shown that other indications are favourable towards the moving of St. Bartholomew's from its present site, this must be an additional reason for taking this course. If it could be guaranteed that the patched-up hospital, which will be the result of partially rebuilding St. Bart's on the present site, would combine a maximum efficiency with a minimum waste of labour and money, few, perhaps, would complain; but it is apparent that by retaining the ancient ward blocks, as it is intended to do, the maximum efficiency cannot be attained. On the contrary, it is as certain as that night follows day that in about twenty years the site, including all the buildings upon it, will probably have to be evacuated, and an entirely new hospital built, unless the staff and the Governors are prepared to permit the high reputation of St. Bartholomew's Hospital to totter to its fall, amidst the reprobation of a succeeding generation, who may then point to the action of the present Governors in refusing to open their eyes to plain facts, as one of the least creditable and most harmful episodes in the history of one of the greatest hospitals which this or any other nation has ever possessed. Further still, no one who has a right to speak with authority can fail to realise and admit, at any rate privately, that in view of the ascertained facts in regard to hospital buildings and modern methods of treatment, the continued occupancy of the old wards, whatever may be done in the way of reconstruction, by cases suffering from open wounds, is, to say the least, open to serious objection, and that there are probably a number of the most able surgeons in the country, who would decline to permit their patients in the ordinary course of their work to be retained in such wards, unless the strongest compulsion was brought to bear and the plea of absolute necessity raised to justify such a proceeding. At the moment no such necessity can be urged by the Governors or the staff of St. Bartholomew's Hospital, and that is why we most earnestly urge every man among them to bring his mind and conscience to bear upon the decision which has to be taken, as to whether or no the present crisis in the history of St. Bartholomew's Hospital shall be turned in such a way as to promote to the utmost the reputation and welfare of this great institution, or whether it shall be lost to its lasting injury and disrepute. In the latter case it is unquestionable that an amount of avoidable risk and unnecessary suffering must be entailed upon a large number of patients who will be received into the wards of this our oldest hospital, and that the measure of this avoidable misery will tend to increase year by year during each of the years that the tinkered and patched-up buildings are continued for hospital purposes. We are confident, that if the public realise it rests with them whether St. Bartholomew's Hospital shall continue on the present site, or whether it shall be moved to a new site where its work can be done with immeasurably greater advantage to the patients, and an increase to the reputation both of the hospital and the great medical school attached to it, they will decline as a matter of fact to subscribe £300,000 for any such purpose as the tinkering up of an old building. This would be bad business for the present generation to enter upon *vis-a-vis* with the assured certainty that their children will in such a case then be asked to give three times the money now raised to get St. Bartholomew's Hospital out of the mess to which it was reduced by the co-operation of the public with the Governors in the year 1903. We take it therefore, that when the actual facts and situation are properly understood and thoroughly realised, the Governors of St. Bartholomew's Hospital and the public must determine that it would be unjustifiable to expend hundreds of thousands of pounds on buildings on the present site, because to do so is economically indefensible, whilst it cannot be upheld on the grounds of humanity, for the tendency of such a course must be to subject the patients to many avoidable risks which would not attach to a new hospital on a new site.

The Kernel of the Whole Matter: A Question of Site.

This brings us to the kernel of the whole matter. Can St. Bartholomew's Hospital purchase a new site in a locality where it will have the fullest facilities for conducting the whole of its work, not only as a great hospital, but as a great medical school? Last week we gave a plan which shows a site at St. Luke's, which, we understand, is quite acceptable to the surgical staff of St. Bartholomew's. Sir Trevor Lawrence, in his letter to the *Times*, does not meet this question of site at all. He merely shelves it by an intimation that all the site contained in the area referred to is not at present the property of the hospital, and that some of it is let under leases with more than twenty years unexpired. We have made careful inquiries and we have reason to believe that these, and indeed all the leases and freeholds of the site referred to, might be obtained upon reasonable terms, so that the whole site could be acquired and placed in the possession of the hospital authorities, say, within two years' time. We must therefore assume for practical purposes that the St. Luke's site is in reality available for the governors of St. Bartholomew's Hospital if they seriously wish to entertain it. Failing this there is a site of equal extent and equal suitability in another part of London, of which we have plans, which may be obtained ready for the builder to immediately commence operations for a sum of from £250,000 to £300,000. We do not mention another site to-day because we must urge upon the treasurer and governors the importance of their looking at the St. Luke's site very carefully and thoroughly before they determine to issue an appeal to the public, or to perpetuate all the disadvantages and injury which may result to St. Bartholomew's Hospital by adopting the proposal to reconstruct the existing buildings, and erect new ones on the land adjacent to the present site. To do so would be to consent to carry on the work of the hospital under conditions infinitely less favourable than those the Governors can provide. The vast expenditure (£550,000) the retention of the old site entails on the Governors is economically indefensible, unless or until they satisfy themselves and the public, by publishing facts demonstrating, not only that the St. Luke's site cannot be utilised, but that there is no other site in the metropolitan area which can be acquired and built upon, without greater injury to St. Bartholomew's Hospital, than must result to that institution should the present proposal be carried out on the lines foreshadowed in the treasurer's letter, published on page 6 of the *Times* of the 12th inst.

Is natural reverence and affection for an old building to be permitted to outweigh every other consideration, and to imperil the reputation and usefulness of a great hospital? We cannot credit such a calamity as a result of the present discussion. Nor is it necessary. For sentiment may be indulged in so far as the existing site is concerned by retaining upon it accommodation for, say, 100 beds, as an outpost hospital to the larger St. Bartholomew's Hospital on the St. Luke's site, so that the traditions of this ancient foundation may be preserved through the centuries, and every necesary accommodation provided at Smithfield to fully meet the needs of the diminishing population of the districts immediately surrounding the old site.

